# AGEISM IS NOT A CATCH-ALL: THE INTERRELATEDNESS OF AGEISM AND ABLEISM

**DOI:** 10.1093/geroni/igad104.1208

**Published:** 2023-12-21

**Authors:** Tracey Gendron, Camp Alyssa, Amateau Gigi, Mullen Mia, Jacobs Kristen, Jenny Inker, Sarah Marrs

**Affiliations:** Virginia Commonwealth University, Richmond, Virginia, United States; Virginia Commonwealth University, Richmond, Virginia, United States; Virginia Commonwealth University, Richmond, Virginia, United States; Leading Age National, Washington, District of Columbia, United States; Leading Age National, Washinton, District of Columbia, United States; Virginia Commonwealth University, Richmond, Virginia, United States; Virginia Commonwealth University, Richmond, Virginia, United States

## Abstract

Ageism represents discrimination based on age, while ableism represents discrimination toward people with disabilities. Given the natural occurrence of physical decline accompanying aging, it is essential to explore how prejudice toward disability intersects with fear about aging. 913 individuals responded to a survey on ageism and ableism. Multiple regression with internalized ageism as the criterion variable was significant F(7, 705) = 81.71, p <.001. Overall, the regression model explained 44.8% of the variance in internalized ageism with significance for age (t(705) = -5.45, p < .001), relational ageism (t(705) = 11.72, p < .001), affinity for older people (t(705) = -2.77, p = .006), ableism for physical disability (t(705) = 6.88, p < .001), and ableism for cognitive disability (t(705) = -2.86, p = .004), Multiple regression with relational ageism as the criterion variable was significant F(7, 705) = 88.12, p <.001. Overall, the regression model explained 46.7% of the variance in relational ageism with a significant for affinity for older people (t(705) = -3.80, p < .001), internalized ageism (t(705) = 11.72, p < .001), relational ableism (t(705) = 6.73, p < .001), ableism for cognitive disability (t(705) = 2.15, p = .007), and ableism for sensory disability (t(705) = -2.78, p = .006). Examining the intersection between ageism and ableism represents the next critical juncture to increasing our understanding of developing effective anti-ageism interventions that address the root anxieties influencing attitudes about aging.

